# The Law and Economics of Generative AI and Copyright: A Primer to Core Challenges for Our Digital Future

**DOI:** 10.1515/rne-2025-0039

**Published:** 2025-11-06

**Authors:** Zachary Cooper, Bertin Martens, Christian Peukert, Volker Stocker

**Affiliations:** 1190 VU Amsterdam, Amsterdam, Netherlands; Bruegel, Brussels, Belgium; Weizenbaum Institut & TU Berlin, Berlin, Germany; HEC Lausanne, Lausanne, Switzerland; Tilburg University, Netherlands; Weizenbaum Institut, Berlin, Germany

**Keywords:** regulation, AI, copyright

## Abstract

Generative AI (GenAI) systems raise fundamental challenges for copyright law at both the input and output stages. On the input side, legal uncertainty surrounds the large-scale scraping of copyrighted data for model training, with divergent rules across jurisdictions and limited transparency on how data is sourced. On the output side, courts struggle to determine when AI-assisted creations are sufficiently human to merit protection, leading to inconsistent or unclear legal outcomes. This paper outlines the “AI copyright conundrum” and examines its impact on the incentives to create, the accessibility of high-quality datasets, and the sustainability of cultural production. We discuss policy options and open questions for research.

## 1 Introduction

Intellectual property rights have emerged as one of the most hotly debated topics surrounding generative AI (GenAI), capturing the attention not only of those industries most affected by the impending uncertain change, but also of policymakers, researchers, and the general public. There are two main areas of contention. The *first* is over the data used to train AI models. Debates continue to rage over which data is used for model training and whether the data used to train models is collected and processed in a legitimate manner. GenAI output is inextricably linked to data input. Data can be subject to copyright protection, which grants its rightsholders (nearly) exclusive rights to control reproduction and reuse. In turn, some rightsholders are unhappy that models have been trained with their works without their consent. Additionally, data access may be unevenly distributed, as some individuals or entities own stocks of data or can tap into data flows, while others are unable to do so (Azoulay et al. 2024). The *second* area concerns the outputs that GenAI models produce and whether they can be subject to copyright protection. Tussles have emerged over the role and necessary degree of human involvement in the content production process. Moreover, GenAI output may rival human creations such as photos, illustrations, books, music, videos, or news.

The debate about intellectual property in AI highlights that data is a crucial fuel for GenAI in increasingly digital economies. This is reflected in foundation models that fuel modern GenAI applications and agents. Data needs and resource demands, as well as efficiencies in training, can vary across different models. This is also true when distinguishing between various stages of model training. While model pre-training typically involves vast amounts of data and is the most compute-intensive step, post-training, as used for model fine-tuning, has significantly lower compute demands. For AI inference and the delivery of AI services, such as AI agents, lower response times and real-time access to data may be required, thus posing very different demands in terms of compute and connectivity than model training (e.g. Ivanov 2024).

Despite its copyright challenges, AI is becoming ubiquitous. GenAI continues to evolve, with regular new or enhanced models, systems, and tools, enabling a litany of expanded uses. Moreover, beyond chat interfaces like ChatGPT, Claude, or Gemini that have gained hundreds of millions of users in a remarkably short time and allow end-users to quickly generate text, images, music, and video, GenAI is becoming increasingly integrated into a wider range of services, including those already used by hundreds of millions of end-users pre-GenAI. Examples of the latter are Microsoft’s integration of OpenAI’s ChatGPT into their Microsoft 365 Copilot which is embedded in Microsoft Office (Spataro 2023, 2025); Apple’s integration of OpenAI’s GPT and potentially soon Google’s Gemini into their Apple Intelligence (Mauran 2025); and Meta’s integration of Meta AI into WhatsApp, Instagram, and Facebook (Meta 2025). While this raises questions about access and ownership of compute and data, it also raises questions about the copyrightability of content produced with (the help of) GenAI. Beyond model transparency, knowing when, how, and to what extent we confront GenAI is becoming increasingly challenging – in many cases, we will lack awareness or the ability to detect it (e.g. Cooper 2025; Cooper et al. 2025b). In fact, diffusion processes are complex, and tracking, tracing, and understanding individual use along with the societal and economic impact of GenAI is non-trivial.

Arguably, these are exciting times for researchers. However, these are also challenging times for law and policymakers. GenAI is reshaping the fabric of business and technological relationships and (inter)dependencies that underlie industry structures and value chains, creating tensions and challenging traditional business models and industry hierarchies (e.g. Stocker and Lehr 2025; Hagiu and Wright 2025). The stakes are high, as is stakeholder involvement. Beyond the participation of market and industry actors whose business models and opportunities are at stake, considerations in reforming intellectual property rights regimes are arguably complicated by the global geopolitical tensions that have emerged in the race towards AI leadership between the US, the EU, and China.

In this short article, we will briefly outline and discuss the core challenges related to GenAI and copyright from a law and economics perspective. In unpacking the multiple facets of the AI copyright conundrum, we explore both input-side and output-side issues to derive policy implications and avenues for future research.

## 2 Contextualizing GenAI Model Training and Output

The AI value chain is often represented in vertical stacks of hardware and software components. Within these stacks, technical and business relationships between different entities are complex and evolving. Beyond high-skilled human labor (e.g. software engineers) and digital infrastructure resources (e.g. GPU-fueled cloud compute), data has emerged as a critical enabler of the models, systems, and tools we commonly refer to as GenAI. Data access and ownership are important to consider, both to assess model capabilities, evolution, and competition, as well as to evaluate model outputs. Data shapes the behavior of AI systems at multiple stages: during model development, refinement, and use. These stages include pretraining on large datasets to create general-purpose foundation models, post-training techniques to adapt models for specific tasks or improve performance (such as alignment, reasoning, efficiency, or integration), in-use conditioning through prompts and in-context learning, or input/output filtering (e.g. Ohm 2024; Tie et al. 2025). Human feedback can play a role throughout, especially during post-training, where models are often fine-tuned or aligned using reinforcement learning with human input (see, e.g. New York Times 2023). Together, these processes show how data flows influence not only how models are built but also how they function in real-world applications. This continuum, from training to deployment, raises important questions about data access, ownership, and intellectual property at each step.

AI systems create output (that represents new data) based on the transformation of inputs (including ingested data). Many of the most widely adopted AI tools generate outputs in response to user requests based on chat interfaces or APIs. With agentic AI, AI systems gain novel capabilities to execute a broad range of (complex) tasks more autonomously - also by using different data as inputs where needed to perform specific tasks (e.g. by accessing users’ web browsers) (e.g. Cloudflare n.d.). Notably, in-context learning yields opportunities for users to ‘steer’ or personalize model output (see, e.g. Batzner et al. 2024). While the degree to which this is possible is arguably influenced by the AI system itself, along with user skill and input, human input is relevant at all stages of model training, including the data ingested into the model, and evidently also in model use. This needs to be factored into evaluations of the role of humans in the loop.

Many of the most widely adopted and used AI models are rather opaque (Bommasani et al. 2024). While this is partially technology-inherent, opacity is sometimes part of business strategy. AI companies often maintain confidentiality over the details of the data, compute, methods, mitigations, or human labor used or employed. However, all of these aspects represent important information for ecosystem stakeholders – for example, in auditing regulatory compliance (e.g. Bommasani et al. 2024; Lehr and Stocker 2024; Stocker and Lehr 2025). The lack of transparency creates a set of questions. For example, without precise knowledge of the data ingested and how it is used for model training, enforcing intellectual property rights becomes difficult, which creates legal uncertainty for all parties involved.

## 3 The AI Copyright Conundrum

Generative AI challenges copyright law at both ends: the use of protected content for training (*input*) and the protectability of AI-assisted works (*output*). On the input side, legal uncertainty around data scraping and licensing threatens both the reuse of existing datasets and the incentives to produce new data. On the output side, courts struggle to define when AI-generated content qualifies as “human-authored,” leading to inconsistent protections. The next section examines both dimensions of the AI copyright conundrum.

### 3.1 Input Side: Access to Data

Access to data is the lifeblood of GenAI development. Yet the legal and economic institutions governing this access are fractured, outdated, and increasingly contested.

Copyright’s traditional economic rationale is to encourage creative production by granting exclusive rights to creators - on the premise that, without such protection, no rational actor would invest in the costly and uncertain process of creation (Hurt and Schuchman 1966; Ohly and Klippel 2007; Klass et al. 2021; Drahos and Braithwaite 2002). These exclusive rights, however, are in principle licensable, allowing for follow-on innovation.

Modern copyright law, however, was not built to govern machine learning at scale. AI models consume enormous volumes of text, images, and code, often scraped from the public web, raising fundamental questions about what constitutes lawful reuse (Samuelson 2023). Currently, there is no globally accepted legal framework governing how GenAI systems can acquire and utilize training data. Instead, developers must navigate a fragmented global landscape.

In the United States, AI developers rely on the doctrine of fair use, but this legal foundation is unsettled. A US Copyright Office report expressed doubts about the applicability of fair use when AI models train on copyrighted works to produce content that competes with these works in existing markets (USCO 2 2025b). It may take several years before all of the ongoing litigation (such as Getty v. Stability AI) reaches a conclusion as to when scraping and repurposing copyrighted content for AI training will constitute fair use. At the time of writing, there are dozens of lawsuits filed against AI companies still pending in the US. Yet recently, two judges issued orders finding both Anthropic and Meta’s use of books to train their models to be fair use (see: Bartz v. Anthropic PBC (3:24-cv-05417) District Court, N.D. California & Kadrey v. Meta Platforms, Inc. (3:23-cv-03417) District Court, N.D. California). Still, Judge Alsup found Anthropic’s initial downloading of the books prior to training to be copyright infringing (resulting in the $1.5 billion largest copyright recovery of all time), casting doubt on how Anthropic could legally create a database to train on in the first place, while Judge Chhabria made clear that he believed that generally training on protected works would be illegal, having found fair use because “these plaintiffs made the wrong arguments”. In the meantime, President Trump’s rhetoric at a “Winning the AI Race” summit in July clearly expressed his preference for permissive copyright laws in order to better compete against China.

The European Union, which does not have fair use, has rather codified text and data mining (TDM) exceptions under Articles 3 and 4 of the Copyright in the Digital Single Market Directive. However, Article 4 allows rights holders to opt out – as many now do (Longpre et al. 2024b), via metadata or contractual terms of service. The AI Act confirms the applicability of these articles to AI model training. The Code of Practice for the implementation of the AI Act goes a step further and requires AI developers to comply with robot.txt and other machine-readable opt-out protocols but also to ensure that these opt-outs do not negatively affect findability of content by search engines. Developers should mitigate risks that a model memorizes copyrighted training content and produces copyright-infringing outputs. A recent study into memorisation in open weight models had mixed results - finding significant memorization within the models, yet also finding significant variation between different models and different copyrighted works (Cooper et al. 2025a). However, collection of infringing data inputs is on the rise as opt-outs are increasingly ignored by AI real-time data retrieval bots that are overwhelming the internet (Longpre 2025; Tollbit 2025; Kim et al. 2025). Web publishers are turning to technical protection measures to stop unauthorized bots.

In effect, these opt-outs can nullify the legal basis for large-scale scraping, making it difficult to identify which data may lawfully be used. The Hamburg ruling on the LAION dataset offered a small but notable development: for the first time, a European court provided guidance on the legality of web-scraped datasets used for AI training. The court suggested that terms of service expressing opt-outs would likely be enforceable under EU law, thus providing some clarity for developers. While this ruling reduces uncertainty in Europe, broader questions about AI training and copyright remain unresolved.

Legal fragmentation, which goes beyond the two exemplary cases of the U.S. and EU, creates compliance risks and market frictions. Developers must consider not only the legal status of each dataset, but also whether rights have been waived, reserved, or conditionally licensed, and how that differs across jurisdictions. As Fiil-Flynn et al. (2022) argue, the lack of globally harmonized and research-compatible TDM exceptions hampers scientific collaboration and innovation alike, and in fact, countries with less restrictive copyright regimes tend to do better in terms of AI innovation (Peukert 2025a). The EU seeks to protect its domestic market by legally doubtful claims about the extra-territorial application of copyright provisions in the AI Act (Quintais 2025).

In the meantime, the absence of legal clarity is not neutral. It raises barriers to entry, privileges actors with large legal teams, and undermines the development of open, accountable AI systems.

Legal uncertainty not only distorts how existing datasets are used, but also how new data is created (Yang and Zhang 2024; Peukert et al. 2024). It puts at risk both the stock and flow of information critical to the development of GenAI. The stock refers to the body of existing, high-quality data available online, including Wikipedia, Common Crawl, GitHub repositories, open-access papers, image archives, and more. This stock is vast, but not infinite. Much of it has already been mined by leading model developers (Jones 2024). Legal uncertainty around copyright and opt-outs now limits the ability to reuse or expand these datasets, especially for smaller players or public-interest projects. More worrying is the flow – the continuous generation of new digital content. This flow is slowing or becoming increasingly privatized (Longpre et al. 2024b): News organizations and image libraries are placing content behind paywalls, terms of service are increasingly restrictive, prohibiting automated scraping, and commercial licensing deals divert fresh content into exclusive arrangements with major AI labs. The result is a narrowing pipeline. If contributors fear that their work will be reused without permission, or fear liability for reusing others’ work, they may stop participating altogether (Peukert et al. 2024). If the flow of diverse, timely, and high-quality data dries up, the training of future models risks becoming stale, unrepresentative, or overfitted to historical trends. More recently, the integration of GenAI into widely deployed applications with direct end-user interfaces (see introduction) emphasizes the trend toward a privatization of data flows.

One might expect that at least the stock of publicly available datasets would be governed by clear rules. Yet this is far from the case. A recent audit of AI data repositories, including Hugging Face and PapersWithCode, revealed that approximately 65 % of datasets had missing, incorrect, or ambiguous licensing information (Longpre et al. 2024a). For example, in LAION-5B, the largest publicly available image dataset, which covers essentially all images on the public internet with contextual information, fewer than 1 in 1,000 datapoints contain license metadata (Shcherbakov et al. 2025). Lack of transparency is not just a compliance headache; it also poses significant risks. Developers may inadvertently infringe on copyright, companies face liability exposure, and researchers are left unsure whether data reuse is legally permissible, which discourages scientific and technical progress (Peukert 2025b).

The causes are manifold: dataset curators often lack incentives or guidance on licensing hygiene; metadata fields are inconsistently applied; and there is no standard enforcement mechanism. While it is clear that full transparency is prohibitively costly to achieve, it is also clear that without better documentation, machine-readable licenses, or provenance tracking, the legal risks multiply, especially for actors without in-house counsel.

In response to these challenges, major AI companies are forging exclusive licensing agreements with content providers. These deals offer legal certainty for the firms involved, but they exacerbate concentration of data access and limit downstream innovation. Content that was once openly accessible now becomes part of proprietary pipelines, locked away from the broader ecosystem.

Furthermore, data with a clear license status (either through opt-in or opt-out) is very likely a non-random and therefore non-representative subset of all potentially accessible data (Shcherbakov et al. 2025). As a result, exclusivity arrangements can render copyright at odds with specific AI regulation, which demands that “training, validation and testing data sets shall be relevant, sufficiently representative, and to the best extent possible, free of errors and complete in view of the intended purpose.” (Art. 10(3)). Exclusivity also interacts with the biases present in existing datasets (Levendowski 2018). The current stock is skewed, with English dominating, perspectives from the Global South underrepresented, and persistent biases regarding culture and gender (Guilbeault et al. 2024). When new data flows are diverted into private deals, the opportunity to correct for these imbalances shrinks. Only those with privileged access can even attempt to mitigate or rebalance the biases embedded in today’s foundation models.

Some view synthetic data as a solution. It can be privacy-preserving, scalable, and tailored to specific domains (Tyagi 2024; Lee 2025). But it is not a panacea. Synthetic data depends on the quality of real data from which it is generated. It cannot produce genuine novelty or social grounding. Moreover, recursive training on synthetic outputs risks drift, loss of relevance, and model collapse (Shumailov et al. 2024). Further, over-reliance on synthetic data may entrench existing biases and errors. Without renewed attention to ensuring access to high-quality, diverse, and legally usable real-world data, the development of robust and trustworthy AI systems remains at risk.

### 3.2 Copyrightability of Output

On the view that copyright exists to encourage creative production that would not occur otherwise, one might reasonably ask why AI-generated works should be excluded - especially if their creation involves non-negligible costs and enables genuinely novel forms of media. However, this is not how this question has been considered. Rather, courts worldwide have sought to determine whether AI-generated works can be considered “human-authored” at all, given the powerful creative contributions of the AI tools themselves (Cooper 2025). Initially, while most national frameworks were silent on the matter (and indeed, still are), frontrunners on the issue, such as the United States, almost entirely agreed that generated outputs should not receive copyright until China first broke the mold in the case of Li v Liu, where the Beijing Internet Court found that the extensive prompting and refinement of the AI tool’s generative parameters reflected enough “intellectual investment” and “personalized expression” on the part of the creator to meet the threshold of “originality” and “intellectual achievement” to receive copyright (Cooper 2025; Li v Liu, 2023).

Since then, the US Copyright Office (USCO)’s former hard-line has at least rhetorically softened, now dichotomising rights between material where “AI tools assist rather than stand in for human creativity” (which receives copyright) and “purely AI-generated material, or material where there is insufficient human control over the expressive elements” (which does not). Importantly, however, the USCO was quick to assert that all current-state outputs of prompting are the latter and therefore uncopyrightable (USCO 1 2025a). There has been no European case law on the issue outside of a single Czech ruling, which denied copyright to an image generated on Dall-E with the reductive reasoning that “[t]he plaintiff did not personally create the work; it was created by artificial intelligence” (Czech Republic Judgment In The Name Of The Republic 2023).

As it stands, then, there is an array of disparate authorship thresholds worldwide, rendering any individual work partly made with AI potentially holding divergent copyright status in each respective national framework, with most of these national authorship thresholds still essentially unclear (Cooper et al. 2025b). This is, of course, far from ideal, as it throws the copyright status of each individual work that has been partly built with GenAI tools into uncertainty – more likely to receive copyright in China, less likely in the USA, and far from certain in either (Cooper 2025).

Worse, given copyright is in many frameworks, such as the US, separatable per element and given GenAI tools are increasingly ubiquitous as mere buttons to be pressed within professional creative workstations for both visual and audio works, this national uncertainty applies to each element within the work. For example, then, anything built using the non-GenAI buttons will be protected, but the partly AI-generated melodies or drums in a song may not have copyright in some countries, depending on how exactly they were created.

Naturally, for such a policy to be robust, a detailed understanding of the entire creative process of each work would be required to delineate which elements are protected by copyright and which are not. As it stands, national frameworks are reliant on artists disclosing AI use. Still, to do so comprehensively, artists would need to maintain a record of every single time they used GenAI tools in their process, despite this record-keeping being entirely in service of them losing rights to their work. One of the authors of this article has previously written of “The Old Hard Drive Debacle”, wherein an artist finds a hard drive full of old work without memory or record of how each work was made (Cooper 2025). In such an instance where they are unable to honestly declare AI use, they are likely to just deny it to receive full rights to their work.

If disclosure does not work, we must rather rely on technological solutions, such as watermarking or provenance mechanisms. Yet, there is currently no robust technological tool that can definitively detect whether any work has or has not incorporated use of a GenAI tool at all, let alone one which could granularly determine on an element-by-element basis exactly where and how each GenAI tool has been used in a detailed enough fashion to make a qualitative assessment as to whether its output should receive copyright or not (Alkhafaji et al. 2020; Begum and Uddin 2020; Chen et al. 2023; Cooper 2025; European Union Intellectual Property Office 2020; Gregory and Vazquez Llorente 2023; Heikkilä 2024; Jacob and Mitra 2015; Sadasivan et al. 2023; Srinivasan 2024).

As it stands, then, there are significant challenges to the sustainability of current-state approaches in hoping to dichotomise AI-generated and non-AI-generated works. Without meaningful reconsideration as to how to maintain and enforce such a policy, alternative frameworks will need to be developed.

### 3.3 Infringing Outputs

It is well established that GenAI tools can be used to create works that are copyright-infringing. On the extreme end, they can create works that are nearly identical to protected works (Cooper and Grimmelmann 2025). Still, liability for such copyright infringement may be complicated, dependent upon both jurisdiction and individual circumstance. Indeed, it is possible for expressly infringing works to be created in circumstances where users had no intention of infringing and where developers have gone to some lengths to try to ensure that their models will not generate copyright-infringing outputs. This raises contentious questions as to the extent to which the current infringement standards rightly or wrongly attribute liability. All this is further complicated by legal exceptions to copyright infringement, such as “fair use” in the USA or “private copying” in the EU, implying that overly filtered models may disallow entirely legal forms of expression, undermining both their general utility and social good.

Yet, the notion of infringing outputs is especially challenged, given the weak similarity thresholds that have led works to be considered derivative works in certain jurisdictions. This means that an output need not necessarily be anywhere near a close replica to be infringing. For example, the controversial US “Blurred Lines” case found similarity in the groove and feel of Robin Thicke’s song to Marvin Gaye’s “Got To Give It Up”, despite the songs being clearly different songs with entirely different melodies, lyrics and chords. If GenAI leads to creative generation *en masse* with vast generation of melodies, chords and images mining out the latent possible space between currently existing works, it may be chaotic to maintain current regimes that consider even marginal incorporation of protected elements (such as a single melody) within larger works as potentially rendering the work as derivative to (and therefore infringing upon) another protected work. Naturally, this is further complicated by the discussion above, wherein copyright is dichotomised between AI-generated and non–AI-generated works without robust means of enforcing this dichotomy.

Still, the more creators use GenAI tools in their process, the more that unintended similarities to protected works may inadvertently appear in their new works (Lemley 2024). Low similarity thresholds for infringement coupled with easy access to generative tools without robust means of dichotomising non/GenAI works inherently incentivizes “copy-mining”, wherein people create as much content as possible in order to have as many rights as possible (Cooper 2025). Thus, at a more foundational level, GenAI tools challenge weaker findings of substantial similarity between works, and in turn, may have a substantial impact on which works are found to be derivative of others.

## 4 Navigating Towards Our Digital Future: Challenges & Policy Considerations

*First*, the legal interpretation of copyright law in an AI context remains uncertain at both the input and output stage, both in the EU (Quintais 2025; Cooper 2025) and in the US (Cooper 2025; USCO 1 2025a). It will likely take years before ongoing and future cases can work their way through the courts and produce more clarity.

*Second*, even if transparency is achieved, content licensing remains an obstacle. For many models, individual licensing may cause prohibitively high transaction costs, let alone license pricing issues, in view of the huge number of small online publishers. As Shcherbakov et al. (2025) show in the exemplary context of the 2 billion images listed in LAION, ten website domains account for 23 % of images. Signing licensing agreements with a few large publishers, as major AI developers have done, therefore leaves a large share of potentially usable data on the table. This can induce a bias in AI training and retrieval datasets: Shcherbakov et al. (2025) show that images with stated license information and images where rightsholders have chosen to opt out of AI training depict semantically different concepts and are of different technical quality (as measured in pixel volume). Some potential solutions often discussed are various forms of collective licensing (Geiger and Iaia 2024; Senftleben 2023). However, feasibility is a huge question mark. The transaction cost burden would simply be shifted from publishers and AI developers to the intermediary organisation that should redistribute the revenue among all publishers. Another solution may then be a hybrid system: distributing royalties where authorship can be reliably attributed, and allocating the remainder - such as to a collective fund that supports small publishers (Output-based remuneration, akin to ideas discussed in Senftleben 2024). In a world where direct compensation for AI usage is impractical at scale, such an approach could help sustain human-made cultural production if this is the broader policy goal.

*Third*, the rapid evolution of AI technology is continuously reshaping the landscape of copyright-relevant practices. While initial concerns focused largely on the collection and use of data for model pre-training, more recent developments have introduced more complex and less transparent dynamics. For instance, knowledge distillation and other reinforcement learning techniques increasingly involve AI models learning from the outputs of other models, raising difficult questions about the origin and copyright status of the underlying content. These secondary uses are often opaque, making it challenging to assess whether and how copyright is implicated. In parallel, the growing reliance on AI agents for real-time data retrieval, aimed at keeping models current and context-aware, has sparked renewed debate over what constitutes lawful access to web-based content (Synodiou 2025). Together, these trends suggest that copyright issues are no longer limited to static training datasets, but extend across the lifecycle of AI systems in ways that remain underexplored in legal doctrine and policy.

The problem of accessing the stock versus the flow of data is best illustrated by an example. Attempts by publishers and EU regulators to prevent AI bots from retrieving online data but still to facilitate search engine data retrieval are understandable from a commercial point of view in order to keep user traffic flowing to websites, but are difficult to cohere entirely with copyright law. They put obstacles on the road to rapid technological convergence between search engines and AI answer engines. More importantly, they keep user search and learning costs artificially high, compared to the reduced costs that AI services can offer to users, and thereby threaten to slow down innovation in society. At the same time, fully unrestricted AI summarization may undermine the business models that fund original content creation: if users increasingly rely on AI summaries and stop visiting the original sources, publishers may no longer have the incentive to invest in high-quality content. This emphasizes the need to find a more balanced solution, one that enables the development of AI tools that lower user search and learning costs, but also ensures that content creators are fairly compensated so that content production can be sustained over time.

*Finally* turning to the output side, it is clear that the regurgitation of training inputs into copyright-infringing outputs appears to violate copyright law. Still, dependent on jurisdiction and circumstance, liability can become complicated. The very nature of GenAI tools may challenge how substantial similarity is determined between protected works, raising larger questions about derivative works in an era where individuals are able to produce works *en masse* with varying (unintended) similarity to one another.
